# Demographic and travel characteristics and self‐reported predeparture SARS‐CoV‐2 testing behavior in air passengers entering the United States from foreign destinations from July to September 2021

**DOI:** 10.1002/iid3.1019

**Published:** 2023-12-20

**Authors:** Anthony Panasci, Shannon Gearhart, Anna Shaum, Arthur J. Simental, Colby Mitchell, Dionne Mitcham, Gilandria Williams, Nadim Shake, Clive Brown, Alida M. Gertz

**Affiliations:** ^1^ Centers for Disease Control and Prevention Division of Global Migration and Quarantine Atlanta Georgia USA; ^2^ Oak Ridge Institute for Science and Education (ORISE) Fellowship Program Oak Ridge Tennessee USA

**Keywords:** air travel, COVID‐19 testing, SARS‐CoV‐2

## Abstract

**Introduction:**

From January 2021 to June 2022, the United States Centers for Disease Control and Prevention required predeparture SARS‐CoV‐2 testing for all air passengers arriving into the United States from a foreign country.

**Methods:**

Using data collected during a surveillance project, we described predeparture testing behavior among a convenience sample of international air passengers entering the United States from July to September 2021 at six US ports of entry. We analyzed pairwise relationships between self‐reported test type, test timing, demographic and travel characteristics, and COVID‐19 vaccination status using chi‐square and Fisher's exact tests.

**Results:**

Participants were more likely to get a NAAT versus antigen test if they identified as non‐Hispanic Asian or Pacific Islander (68.2%, *n* = 173), non‐Hispanic Black (62.6%, *n* = 147), or if they preferred not to report race and ethnicity (60.8%, *n* = 209) when compared to those who identified as non‐Hispanic White (47.1%, *n* = 1086, all *p* < 0.05). Those who identified as Hispanic or Latino (*n* = 671) were less likely to get a NAAT than the non‐Hispanic White group (39.5% vs. 47.1%, *p* < 0.05). Participants arriving in the US from the Americas were less likely to get a NAAT (38.5%, *n* = 871) compared to those arriving from Europe (45.5%, *n* = 1165, *p* < 0.05). Participants who reported receiving their predeparture test 2 days or 3 or more days before departure were more likely to report receiving a NAAT (52.2%, *n* = 879, and 60.2%, *n* = 410, respectively) than those who reported testing within 1 day (41.4%, *n* = 1040, all *p* < 0.001) of departure.

**Discussion:**

Test type was significantly associated with race and ethnicity, departure region, and test timing. Differences likely reflected regional disparities in the availability of tests at the time of the activity. Discrepancies in predeparture test timing and type worldwide may have consequences for the effectiveness and equity of travel requirements in future pandemics.

## Graphical abstract

Using data collected during a pilot surveillance program, this manuscript describes predeparture testing behavior among a convenience sample of international air passengers entering the United States from July to September 2021 at six US ports of entry. We analyzed pairwise relationships between self‐reported test type, test timing, demographic and travel characteristics, and COVID‐19 vaccination status. We found that test type was significantly associated with departure region, likely reflecting the regional disparities in the availability of tests at the time of the activity.

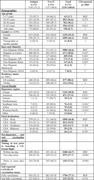

## 1 INTRODUCTION

In January 2021, the United States Centers for Disease Control and Prevention (CDC) issued an Order that required all air passengers aged 2 years or older to present to airlines a negative SARS‐CoV‐2 viral test (nucleic acid amplification test [NAAT] or antigen test) taken no more than 3 days before flight departure, or documentation of recovery from COVID‐19 within the previous 90 days, before boarding a flight to the US.^1^ This Order was subsequently amended in October and December 2021 to shorten the predeparture testing window to 1 day. CDC rescinded the testing Order on June 12, 2022.^2^

There is limited information on the predeparture testing behaviors of international air passengers arriving in the United States during the time the Order was in effect. This report describes the self‐reported predeparture testing behaviors among air passengers who entered the US at various international airports from July to September 2021. There were no vaccination requirements in place at the time this survey was conducted. Understanding the SARS‐CoV‐2 testing behavior of international travelers during this period can help inform the planning and implementation of travel testing policies in response to future emerging infectious diseases.

## 2 METHODS

### 2.1 Setting & population

From July 26 to September 29, 2021, CDC conducted an airport‐based cross‐sectional data capture of international air passengers entering the US at six ports of entry (POEs). Participants were recruited onsite at Miami (MIA), Hartsfield‐Jackson Atlanta (ATL), Washington Dulles (IAD), Boston Logan (BOS), and Los Angeles (LAX) international airports. Additionally, Colorado (CO) residents returning from international air travel, mostly through Denver International Airport (DEN), were recruited remotely via phone or email. Participation was voluntary.

All consenting adult passengers (18 years and older) arriving at the six POEs on US‐bound air conveyances were eligible to participate. Minors (ages 2−17 years) traveling with a parent or legal guardian who provided permission were also eligible to participate.

These data were collected as part of a post‐arrival SARS‐CoV‐2 surveillance testing project. However, the test used in this project was recalled and, thus, the test results are not presented. Given the original intent of the project, those who self‐reported recovery from COVID‐19 within the previous 90 days were excluded because of the potential for false positive results.^3^

### 2.2 Project design

Initial recruitment procedures varied based on recruiting location. Where recruitment was on‐site, CDC quarantine station staff met international arriving passengers at baggage claim. Interested passengers scanned a QR code to verify their eligibility, provided informed consent, and completed a survey via a secure CDC REDCap (Research Electronic Data Capture) weblink.^4^ Where recruitment was remote, project staff emailed international air passengers with Colorado addresses 1 day after their arrival into the US with information on the project and the same survey link. The list of Colorado residents was obtained from the CDC data system in place to provide traveler contact information for air passengers arriving from foreign countries to health departments for the purpose of public health follow‐up. This project design was reviewed and approved by CDC and was conducted consistent with applicable federal law and CDC policy.*

### 2.3 Variables

The survey collected self‐reported demographic, travel, predeparture SARS‐CoV‐2 testing, and COVID‐19 vaccination data. Demographic information included age group, gender, race, and ethnicity. Travel information included departure country, arrival airport, final destination (US state, territory, or other country), and whether the participant was visiting or returning to a residence in the United States. Departure region followed the classification of the World Health Organization (WHO) which divides the world into six regions based on geography and organizational groupings of its Member States. For predeparture testing, participants were asked how many days before traveling to the US (same day of travel, 1, 2 , 3 , or 4 or more days before travel) they had obtained a SARS‐CoV‐2 test and the test type (antigen or NAAT). Testing timing and test type were not verified. For the purpose of analysis, same day of travel and 1 day before travel were collapsed into “Within 1 day,” and 3 days before travel and 4 or more days before travel were collapsed to “3 or more days prior,” to allow for analysis of data in three ordered categories. Of note, testing 4 or more days before departure was not permitted during the survey period.

Self‐reported COVID‐19 vaccination history included the vaccine type, number of doses, and whether the last dose was received more than 2 weeks previously. Only vaccines approved or authorized for emergency use by the US Food and Drug Administration (FDA) or listed by the WHO's Emergency Use Listing Procedure during the project time period were included in the definition of “fully vaccinated” for the purpose of this analysis.^5^ Those self‐reporting immunization with Johnson & Johnson's Janssen vaccine were classified as fully vaccinated if they reported receiving at least one dose and more than 2 weeks had passed since this dose. Those reporting immunization with AstraZeneca/Covishield, Moderna, Pfizer‐BioNTech, Sinopharm, and Sinovac were classified as fully vaccinated if they reported receiving at least two doses and more than 2 weeks had passed since their second dose. Those vaccinated with non‐FDA/WHO authorized vaccines were classified as not fully vaccinated.

### 2.4 Analysis

All data were exported from CDC's REDCap database and analyzed using R version 4.0.4.^6^ We conducted a descriptive analysis of self‐reported demographic and travel characteristics by predeparture test type and timing, employing *χ* ^2^ and Fisher's exact tests with Bonferroni correction to evaluate group differences.

## 3 RESULTS

**Figure 1:**
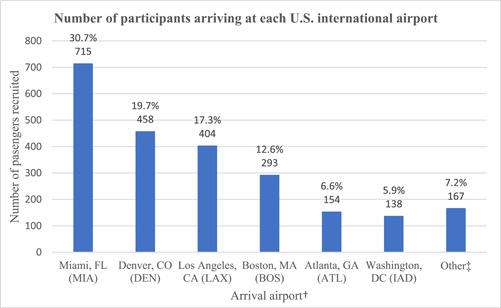
US airport of arrival for a sample of air passengers who participated in an assessment of traveler predeparture COVID‐19 testing behavior, July 26 to September 29, 2021. ^†^Arrival airport was self‐reported. ^‡^Other represents Colorado residents who flew into other airports (recruited after arrival) or other travelers in transit who scanned the QR code on project signage in recruiting airports when connecting domestically to another US airport.

Among 2329 participants, most were aged 18−50 years old (66.7%, *n* = 1554), female (53.5%, *n* = 1229), and of Hispanic or Latino ethnicity (28.8%, *n* = 671) or non‐Hispanic White (46.6%, *n* = 1086) (Table 1). The greatest number of participants reported arriving at MIA (30.7%, *n* = 715), DEN (19.7%, *n* = 458), and LAX (17.3%, *n* = 404) airports (Figure 1). Nearly 90% of participants in our sample arrived from Europe (50.0%, *n* = 1165) or the Americas (37.4%, *n* = 871). One participant whose travel originated in the Americas traveled from Canada; the remainder traveled from Mexico, Central or South America, or the Caribbean (data not shown). Among participants who self‐reported residency status, four in five reported a US residence (80.4%, *n* = 877). Just over half of the participants (51.2%, *n* = 1192) reported their predeparture SARS‐CoV‐2 viral test was by an antigen test. For test timing, more participants reported obtaining their predeparture test within 1 day of boarding a US‐bound flight (44.7%, *n* = 1040), versus 2 (37.7%, *n* = 879) or 3 or more days (17.6%, *n* = 410) prior. Approximately three in four participants reported being fully vaccinated (77.1%, *n* = 1796).

**Table 1:** Self‐reported predeparture SARS‐CoV‐2 test type by demographic characteristics and travel details for a sample of air passengers arriving into the United States from July 26 to September 29, 2021.

	Antigen *n* (%) 1192 (51.2)	NAAT *n* (%) 1137 (48.8)	Total *n* (%) 2329 (100.0)	*χ* ^2^ *p* Value
Demographics				
Age group (years)				
2−17	25 (39.7)	38 (60.3)	63 (2.7)	REF
18−35	451 (52.9)	401 (47.1)	852 (36.6)	0.573
36−50	353 (50.3)	349 (49.7)	702 (30.1)	1
51−65	290 (52.2)	266 (47.8)	556 (23.9)	0.811
≥66	73 (46.8)	83 (53.2)	156 (6.7)	1
Gender (*n* = 2299)				
Female	615 (50.0)	614 (50.0)	1229 (53.5)	REF
Male	558 (52.7)	501 (47.3)	1059 (46.1)	0.663
Transgender or another gender	6 (54.5)	5 (45.5)	11 (0.5)	1
Race and ethnicity				
Non‐Hispanic White	575 (52.9)	511 (47.1)	1086 (46.6)	REF
Hispanic or Latino	406 (60.5)	265 (39.5)	671 (28.8)	0.045^a^
PNTA	82 (39.2)	127 (60.8)	209 (9.0)	0.006^a^
Non‐Hispanic API	55 (31.8)	118 (68.2)	173 (7.4)	<0.001^a^
Non‐Hispanic Black	55 (37.4)	92 (62.6)	147 (6.3)	0.009^a^
Non‐Hispanic multiracial	16 (44.4)	20 (55.6)	36 (1.5)	1^a^
Non‐Hispanic AIAN	3 (42.9)	4 (57.1)	7 (0.3)	1^a^
Residency status (*n* = 1091)				
US resident	468 (53.4)	409 (46.6)	877 (80.4)	REF
Visitor	114 (53.3)	100 (46.7)	214 (19.6)	1
Travel details				
Departure region				
Europe	635 (54.5)	530 (45.5)	1165 (50.0)	REF
Americas	536 (61.5)	335 (38.5)	871 (37.4)	0.032^a^
Eastern Mediterranean	1 (1.3)	77 (98.7)	78 (3.3)	<0.001^a^
Southeast Asia	7 (9.2)	69 (90.8)	76 (3.3)	<0.001^a^
Africa	4 (5.7)	66 (94.3)	70 (3.0)	<0.001^a^
Western Pacific	3 (5.4)	53 (94.6)	56 (2.4)	<0.001^a^
Other	6 (46.2)	7 (53.8)	13 (0.6)	1^a^
Final destination				
USA‐West	579 (53.1)	511 (46.9)	1090 (46.8)	REF
USA‐South	415 (51.1)	397 (48.9)	812 (34.9)	1
USA‐Northeast	154 (45.0)	188 (55.0)	342 (14.7)	0.108
USA‐Midwest	36 (61.0)	23 (39.0)	59 (2.5)	1
Other	8 (30.8)	18 (69.2)	26 (1.1)	0.397
Predeparture test and vaccination status				
Timing of test before boarding a US‐bound flight				
Within 1 day	609 (58.6)	431 (41.4)	1040 (44.7)	REF
Two days prior	420 (47.8)	459 (52.2)	879 (37.7)	<0.001
Three or more days prior	163 (39.8)	247 (60.2)	410 (17.6)	<0.001
Self‐reported COVID‐19 vaccination status				
Fully vaccinated	935 (52.1)	861 (47.9)	1796 (77.1)	REF
Not fully vaccinated	257 (48.2)	276 (51.8)	533 (22.9)	0.131

Abbreviations: AIAN, American Indian or Alaska Native; API, Asian or Pacific Islander; NAAT, nucleic acid amplification test; PNTA, prefer not to answer; WHO, World Health Organization.

^a^ Fisher's exact *p*‐value is provided because *χ* ^2^ test assumptions were breached in at least one 2 × 2 pairwise test.

Race and ethnicity, departure region, and predeparture test timing were significantly associated with predeparture test type. Participants were more likely to get a NAAT versus antigen test if they identified as non‐Hispanic Asian or Pacific Islander (68.2%, *n* = 173), non‐Hispanic Black (62.6%, *n* = 147), or if they preferred not to report race and ethnicity (60.8%, *n* = 209) when compared to those who identified as non‐Hispanic White (47.1%, *n* = 1086, all *p* < 0.05). Those who identified as Hispanic or Latino (*n* = 671) were less likely to get a NAAT than the non‐Hispanic White group (39.5% vs. 47.1%, *p* < 0.05).

Participants arriving in the US from the Americas were less likely to get a NAAT (38.5%, *n* = 871) compared to those arriving from Europe (45.5%, *n* = 1165, *p* < 0.05). In comparison, incoming passengers from the remaining regions were more likely to report getting a NAAT (all >90%, *p* < 0.001). In addition, participants who reported receiving their predeparture test 2 days or 3 or more days before departure were more likely to report receiving a NAAT (52.2%, *n* = 879, and 60.2%, *n* = 410, respectively) than those who reported testing within 1 day (41.4%, *n* = 1040, all *p* < 0.001) of departure. Those who self‐reported Hispanic or Latino ethnicity (vs. non‐Hispanic White), non‐US residency, arriving from the Americas (vs. Europe), and not being fully vaccinated were more likely to obtain a SARS‐CoV‐2 viral test within 1 day of travel versus 2 or more days before travel (Appendix).

## 4 DISCUSSION

Building on mathematical modeling studies estimating the effectiveness of layered mitigation measures on travel‐related SARS‐CoV‐2 transmission,^7^, ^8^ recently published evidence observed that rescission of mandatory predeparture testing in June 2022 was followed by an increase in post‐arrival positivity in pooled samples of international air passengers arriving at Newark Liberty (EWR), John F. Kennedy International (JFK), Hartsfield‐Jackson Atlanta (ATL), and San Francisco (SFO) international airports.^9^ In this cross‐sectional survey of international air passengers entering the US at six US POEs from July to September 2021, we investigated the association between certain passenger characteristics and their self‐reported predeparture SARS‐CoV‐2 testing behaviors.

We found that the type of viral test the passenger reported completing before departure was significantly associated with passengers' race and ethnicity, departure region, and test timing. Departure region likely confounds the relationship between race, ethnicity, and test type. For example, when examining the racial and ethnic patterns for test type in passengers arriving from the Americas, the proportion of antigen tests in each racial and ethnic group was higher than for those same groups overall. Similarly, overall racial and ethnic associations disappear when disaggregating by the Eastern Mediterranean, Southeast Asian, African, and Western Pacific Regions, where almost everyone received a NAAT. This suggests that predeparture test type patterns largely reflect regional availability rather than racial and ethnic preference. For the African, Eastern Mediterranean, Southeast Asian, and Western Pacific regions, observed test type patterns could reflect limited access to antigen tests^10^; however, it is difficult to draw any conclusions about these regions given altogether only 12.6% of participants in this survey reported traveling from one of these areas. The majority of travelers, 87.4%, reported arriving from either Europe or the Americas. Over 98% of the participants who reported getting an antigen test reported departing from Europe or the Americas.

Those who obtained a test closer to their departure date were more likely to report getting an antigen test than a NAAT. This is not surprising as SARS‐CoV‐2 NAATs were more often laboratory based during this time period and often required at least 24 h for processing and reporting.^11^ Those who arrived from the Americas were also more likely to test within 1 day of travel versus 2 or more days before travel than those arriving from Europe, which could reflect comparatively higher use of antigen tests in these countries compared to Europe, where NAAT might have been more readily available at the time of our survey. There were no significant differences in test type across US residency or self‐reported vaccination status; however, passengers who were visiting or not fully vaccinated were more likely to get tested closer to departure than those returning home or those who were fully vaccinated, respectively.

This project has several limitations. The convenience sample was imbalanced across the six POEs as well as the departure locations and did not represent all US‐bound air passengers who arrived during the survey period. Both of these limitations could have greatly impacted the sample distributions for race, ethnicity, and departure region. Additionally, test timing, test type, and vaccination history may suffer from recall bias (all data were self‐reported) and/or response bias (some travelers may not have known the difference between antigen tests and NAATs). Also, two of the variables had incomplete data (residency status and gender). The findings also reflected a period when air passengers were allowed 3 days before departure to complete the required testing rather than later periods when the testing window was shortened to 1 day.

The findings here can help to guide decision‐making for predeparture travel requirements during future public health emergencies. Discrepancies in predeparture test timing and type worldwide may have consequences for the effectiveness and equity of travel requirements in future pandemics.

## AUTHOR CONTRIBUTIONS

**Anthony Panasci**: Investigation (supporting), project administration (supporting), resources (supporting), visualization (lead), writing—original draft preparation (lead), writing—review and editing (lead). **Shannon Gearhart**: Conceptualization (lead), data curation (lead), formal analysis (supporting), investigation (lead), methodology (lead), project administration (lead), resources (lead), supervision (lead), validation (lead), visualization (lead), writing—original draft preparation (lead), writing—review and editing (lead). **Anna Shaum**: Data curation (equal), investigation (supporting), methodology (supporting), project administration (supporting), resources (supporting), supervision (supporting), visualization (supporting), writing—original draft preparation (supporting), writing—review and editing (supporting). **Arthur J. Simental**: Data curation (equal), investigation (supporting), methodology (supporting), project administration (supporting), resources (lead), software (lead), supervision (supporting), writing—original draft preparation (supporting), writing—review and editing (supporting). **Colby Mitchell**: Investigation (supporting), project administration (supporting), resources (supporting), writing—original draft preparation (supporting), writing—review and editing (supporting). **Dionne Mitcham**: Investigation (supporting), project administration (supporting), resources (supporting), writing—original draft preparation (supporting), writing—review and editing (supporting). **Gilandria Williams**: Investigation (supporting), project administration (supporting), resources (supporting), writing—original draft preparation (supporting), writing—review and editing (supporting). **Nadim Shake**: Investigation (supporting), project administration (supporting), resources (supporting), writing—review and editing (supporting). **Clive Brown**: Conceptualization (lead), formal analysis (supporting), investigation (supporting), supervision (supporting), writing—original draft preparation (supporting), writing—review and editing (supporting). **Alida M. Gertz**: Conceptualization (lead), data curation (lead), formal analysis (lead), investigation (lead), methodology (lead), project administration (lead), resources (lead), supervision (lead), validation (lead), visualization (lead), writing—original draft preparation (lead), writing—review and editing (lead).

## CONFLICT OF INTEREST STATEMENT

The authors declare no conflict of interest.

## ETHICS STATEMENT

This project design was reviewed and approved by the Centers for Disease Control and Prevention (CDC) and was conducted consistent with applicable federal law and CDC policy (45 C.F.R. part 46, 21 C.F.R. part 56; 42 U.S.C. §241(d); 5 U.S.C. §552a; 44 U.S.C. §3501 et seq). Informed consent was obtained for all participants; parental permission was obtained for participating minors.

## Data Availability

The data that support the findings of this survey are available on request from the corresponding author.

## Footnotes

* 45 C.F.R. part 46, 21 C.F.R. part 56; 42 U.S.C. §241(d); 5 U.S.C. §552a; 44 U.S.C. §3501 et seq.

## APPENDIX A

Table A1

**Table A1:** Self‐reported predeparture SARS‐CoV‐2 test timing by demographic characteristics and travel details for a sample of air passengers arriving into the United States from July 26 to September 29, 2021.

	Within 1 day of travel *n* (%) 1040 (44.7)	Two or more days before travel *n* (%) 1289 (55.3)	Total *n* (%) 2329 (100.0)	*χ* ^2^ *p* Value
Demographics				
Age group (years)				
2−17	34 (54.0)	29 (46.0)	63 (2.7)	REF
18−35	407 (47.8)	445 (52.2)	852 (36.6)	1
36−50	334 (47.6)	368 (52.4)	702 (30.1)	1
51−65	212 (38.1)	344 (61.9)	556 (23.9)	0.215
≥66	53 (34.0)	103 (66.0)	156 (6.7)	0.098
Gender (*n* = 2299)				
Female	541 (44.0)	688 (56.0)	1229 (53.5)	REF
Male	479 (45.2)	580 (54.8)	1059 (46.1)	1^a^
Transgender or another gender	3 (27.3)	8 (72.7)	11 (0.5)	1^a^
Race and ethnicity				
Non‐Hispanic White	394 (36.3)	692 (63.7)	1086 (46.6)	REF
Hispanic or Latino	387 (57.7)	284 (42.3)	671 (28.8)	<0.001^a^
PNTA	97 (46.4)	112 (53.6)	209 (9.0)	0.135^a^
Non‐Hispanic API	80 (46.2)	93 (53.8)	173 (7.4)	0.298^a^
Non‐Hispanic Black	63 (42.9)	84 (57.1)	147 (6.3)	1^a^
Non‐Hispanic multiracial	18 (50.0)	18 (50.0)	36 (1.5)	1^a^
Non‐Hispanic AIAN	1 (14.3)	6 (85.7)	7 (0.3)	1^a^
Residency status (*n* = 1138)				
US resident	335 (38.2)	542 (61.8)	877 (80.4)	REF
Visitor	113 (52.8)	101 (47.2)	214 (19.6)	<0.001
Travel details				
Departure region				
Europe	446 (38.3)	719 (61.7)	1165 (50.0)	REF
Americas	454 (52.1)	417 (47.9)	871 (37.4)	<0.001
Eastern Mediterranean	37 (47.4)	41 (52.6)	78 (3.3)	1
Southeast Asia	34 (44.7)	42 (55.3)	76 (3.3)	1
Africa	36 (51.4)	34 (48.6)	70 (3.0)	0.821
Western Pacific	28 (50.0)	28 (50.0)	56 (2.4)	1
Other	5 (38.5)	8 (61.5)	13 (0.6)	1
Final destination				
USA‐West	431 (39.5)	656 (60.5)	1090 (46.8)	REF
USA‐South	421 (51.8)	391 (48.2)	812 (34.9)	<0.001
USA‐Northeast	144 (42.1)	198 (57.9)	342 (14.7)	1
USA‐Midwest	25 (42.4)	34 (57.6)	59 (2.5)	1
Other	19 (73.1)	7 (26.9)	26 (1.1)	0.012
Vaccination status				
Self‐reported COVID‐19 vaccination status				
Fully vaccinated	757 (42.1)	1039 (57.9)	1796 (77.1)	REF
Not fully vaccinated	283 (53.1)	250 (46.9)	533 (22.9)	<0.001

Abbreviations: AIAN, American Indian or Alaska Native; API, Asian or Pacific Islander; NAAT, nucleic acid amplification test; PNTA, prefer not to answer; WHO, World Health Organization.

^a^ Fisher's exact *p*‐value is provided because *χ* ^2^ test assumptions were breached in at least one 2 × 2 pairwise test.
